# An Unusual Cause of Shoulder Pain: Sternoclavicular Joint Septic Arthritis

**DOI:** 10.7759/cureus.107109

**Published:** 2026-04-15

**Authors:** Jennifer J Price, George Shahin

**Affiliations:** 1 Internal Medicine, Olive View University of California Los Angeles Medical Center, Sylmar, USA

**Keywords:** acute shoulder pain, osteomyelitis, sternoclavicular joint, sternoclavicular joint septic arthritis, surgical resection

## Abstract

Septic arthritis of the sternoclavicular joint (SCJ) is an uncommon location of septic joint infection and is often a diagnostic challenge. Patients may present with insidious, vague symptoms, such as chest pain or shoulder pain, and imaging modalities are often nonspecific, leading to a delay in diagnosis and treatment. We present a case of a young male patient initially presenting with right shoulder pain, but who then returned days later and was diagnosed with SCJ septic arthritis. This patient was treated successfully with SCJ resection and intravenous antibiotics. This case illustrates the difficulty in diagnosing SCJ septic arthritis and how early surgical intervention and medical management can prevent further serious complications.

## Introduction

Sternoclavicular joint (SCJ) septic joint arthritis is a rare diagnosis, accounting for about 1% of all bone and joint infections. In healthy adults, it is less common and has been reported in only 0.5% of all bone and joint infections [1].

The sternoclavicular joint is often overlooked during physical examination. Patients often present with vague chest discomfort or referred neck or shoulder pain in SCJ septic arthritis. It can be difficult to diagnose, as the disease can be insidious in onset, with a median duration of symptoms of 14 days. Failure or delay in diagnosis can lead to serious complications, as 60% of patients who present with SCJ septic arthritis have concomitant bacteremia. Common complications include chest wall abscess, mediastinitis, endocarditis, septic shock, and even death [1,2].

We present a case of a 32-year-old male initially presenting with right shoulder pain, treated conservatively as a shoulder sprain, but then diagnosed at follow-up 3 days later with right SCJ septic arthritis. We highlight the clinical features, diagnostic approach, and medical/surgical management for SCJ septic arthritis, in which prompt intervention improves outcomes and reduces mortality and long-term morbidity.

## Case presentation

A 32-year-old male with a history of gout, uncontrolled diabetes (glycated hemoglobin (HbA1c) 8.8%), and hypertension presented to the urgent care (UC) with right proximal shoulder pain for 1 day. He denied trauma or injury to the affected shoulder; however, he endorsed physical work, lifting heavy objects. On physical exam, right clavicular swelling and tenderness were noted; however, X-ray of the right shoulder and clavicle showed osteoarthritis and no evidence of acute fracture or dislocation. He was sent home with conservative management for a possible shoulder sprain with rest, ice/heat packs, and Tylenol as needed for pain.

This patient returned three days later to the UC, as he had worsening right shoulder and clavicular pain. On physical exam, he had tenderness, erythema, and swelling of his right sternoclavicular joint, as well as a limited range of motion of his right shoulder due to pain. Vital signs demonstrated that the patient was afebrile, and heart rate and blood pressure were within normal limits. Labs showed an elevated white blood cell (WBC) count of 15.0 K/cumm, C-reactive protein (CRP) elevated to 316.6, HIV non-reactive, and uric acid elevated at 6.3 mg/dl.

**Figure 1:**
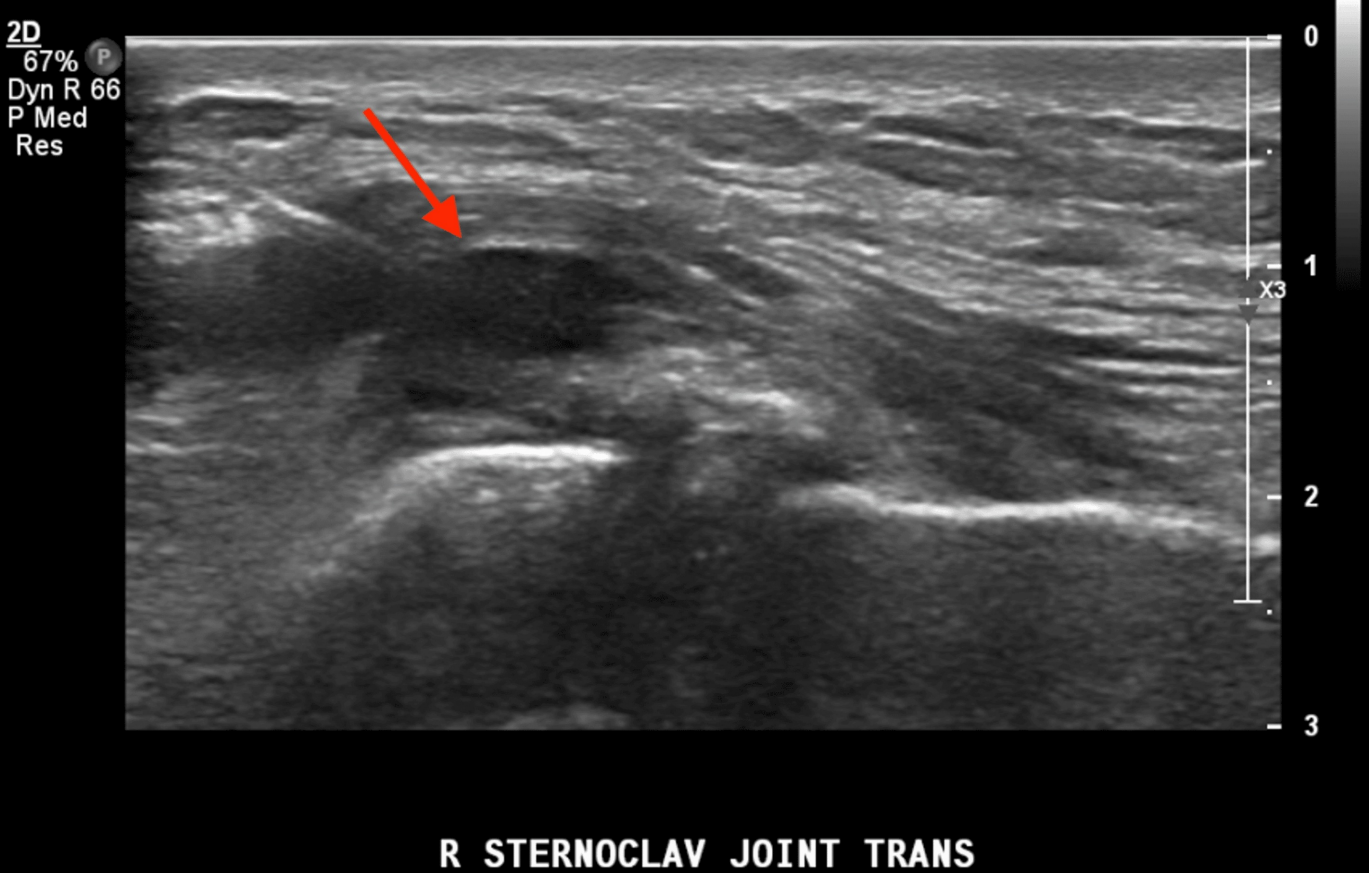
Ultrasound aspiration of the right sternoclavicular joint (red arrow)

Ultrasound (US) of the right shoulder and right sternoclavicular joint showed joint effusion and soft tissue swelling around the right sternoclavicular joint. The right shoulder was otherwise normal; inflammatory arthritis could not be excluded. Given his prior history of gout, there was suspicion of a possible gout flare; therefore, a diagnostic ultrasound with aspiration was then performed of the right SCJ joint, as shown in Figure 1, which yielded a few ml of purulent discharge.

**Figure 2:**
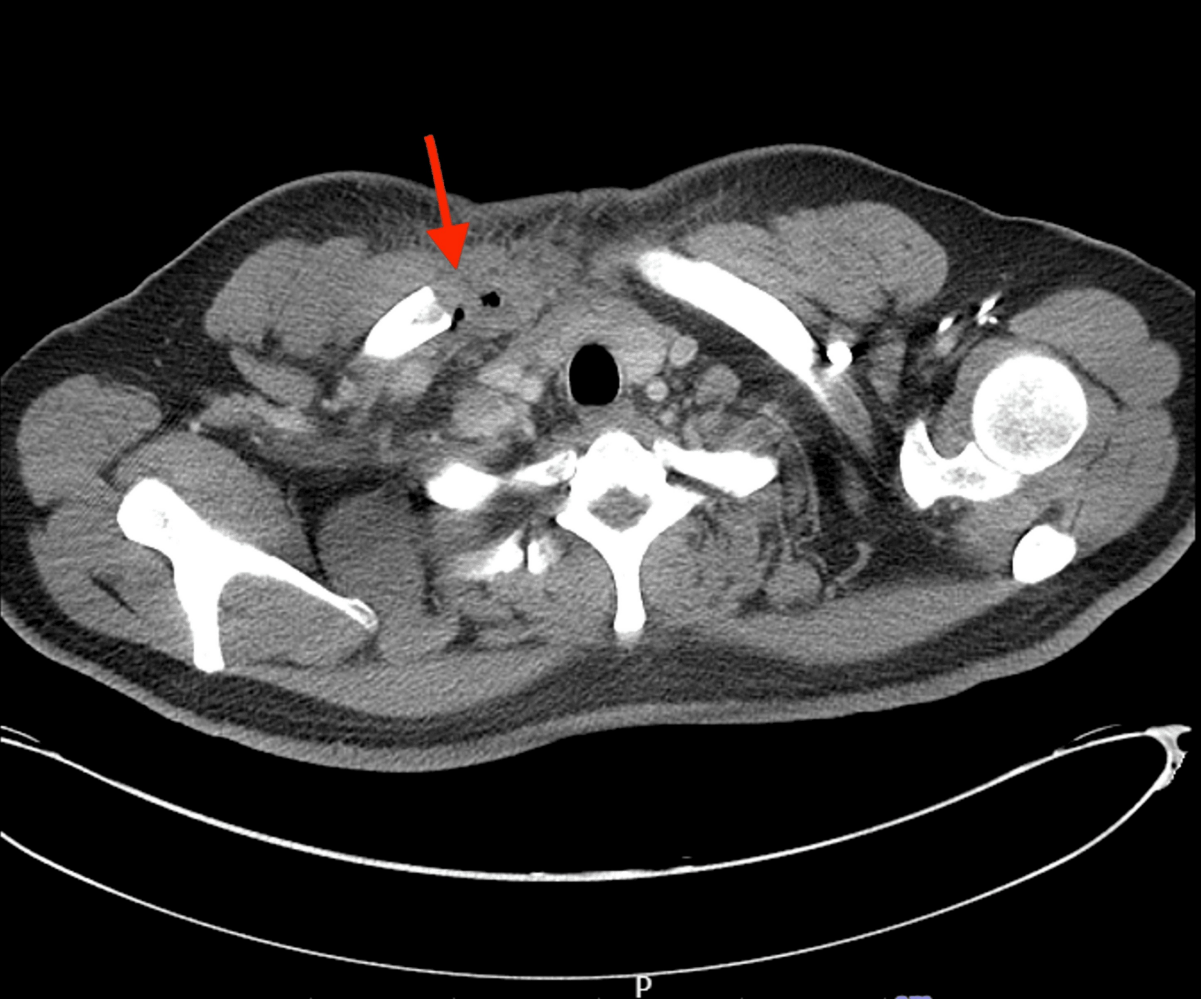
CT scan showing right sternoclavicular joint effusion, associated stranding, bony erosions concerning for septic joint arthritis and osteomyelitis (red arrow)

**Figure 3:**
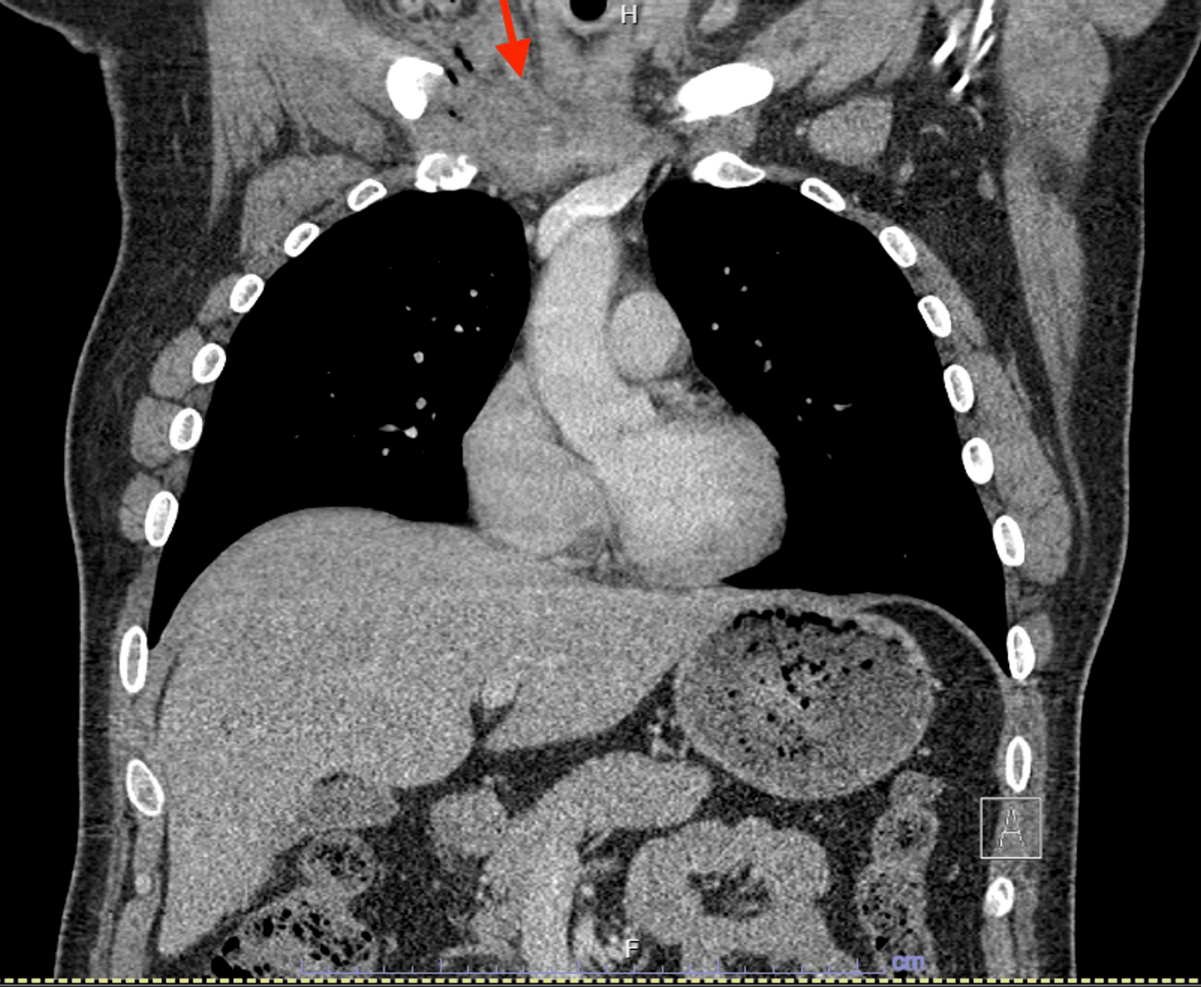
CT scan of the right sternoclavicular joint effusion showing possible extension into the mediastinum (red arrow)

CT thorax was also performed and showed a right SCJ effusion with capsular thickening, associated stranding, and bony erosions extending into the adjacent chest wall and mediastinum, concerning for a right SCJ septic arthritis and osteomyelitis, as seen in Figures 2-3.

Given these findings, the patient was admitted for intravenous (IV) antibiotics and thoracic surgery consultation. Thoracic surgery performed a right clavicular head resection with extensive regional debridement. Blood cultures and right SCJ fluid cultures were positive for methicillin-sensitive *Staphylococcus aureus* (MSSA), concerning for concomitant endocarditis. The patient denied IV drug use and did not have an indwelling catheter.

Infectious Disease (ID) was consulted with a plan to narrow from broad-spectrum antibiotics, IV vancomycin and ceftriaxone, to IV cefazolin given culture sensitivities. The transesophageal echocardiogram (TEE) was negative for endocarditis. The patient was discharged with a four-week course of IV cefazolin through the peripherally inserted central catheter (PICC) line and was treated successfully with complete resolution of infection.

## Discussion

In a study of 967 patients with bone and joint infections by Bar-Natan et al., only 11 were found to have SCJ septic arthritis, accounting for about 1% of bone and joint infections. This diagnosis is fairly uncommon and rare, especially in otherwise healthy adults [1].

In a review of 180 cases of SCJ septic arthritis by Ross et al., the mean age of patients was 45 years, younger than in other types of septic arthritis. Other risk factors include male gender (73%), intravenous drug use (21%), and diabetes mellitus (13%) [2]. Infection of the joint can occur via direct inoculation or, more commonly, hematogenous spread via the bloodstream; therefore, it presents more commonly in patients with IV drug use [3]. No risk factors for infection were reported in almost a quarter of patients [2]. Our patient had some demographics consistent with those commonly presenting with SCJ septic arthritis, such as diabetes and gender; however, he denied IV drug use and was younger than is characteristic of this patient population. 

Clinical symptoms can be insidious, as the SCJ is a non-distensible space that allows effusions to progress slowly. The patient can present with chest pain radiating to the SCJ (78%); however, referred pain from the shoulder (24%) or neck (2%) is also a presenting symptom and can lead to misdiagnosis [4], such as in our patient, who was initially diagnosed with a shoulder sprain. Generally, septic arthritis has a median of 3 days of symptoms at presentation; however, for SCJ septic arthritis, the median duration of symptoms is 14 days. This also explains why there is often a high likelihood of osteomyelitis (55%), as infection is well-established by the time of diagnosis.

Fever can be low grade and is present in only 65% of patients with SCJ septic arthritis [2]. Laboratory markers can be nonspecific; elevated WBC count, ESR, and CRP can be elevated in gout and other systemic illnesses or infections. Our patient had an elevated WBC and CRP; however, it was still difficult to distinguish between a diagnosis of gout or SCJ septic arthritis. Blood cultures can be helpful, as up to 60% of patients are bacteremic on presentation; however, the gold standard for diagnosis would be arthrocentesis [3]. Needle aspiration can be difficult due to the small size of the joint and the presence of an intra-articular disc [1]; however, when it can be performed, per Ross et al., fluid cultures are positive in 77% of patients, while 36% of patients have positive cultures from surgical specimens [2].

Initial plain radiography is often unrevealing (85%) [2], but CT scans and MRI are often diagnostic and show radiological features of septic joint osteomyelitis, chest wall abscess, or mediastinitis [5]. Our patient had an initial XR of the shoulder/clavicle that only demonstrated osteoarthritis. It is important to consider other imaging modalities if concerned for SCJ septic arthritis. There have been case reports of the use of US to detect SCJ septic arthritis, which could be helpful when advanced imaging modalities are not available [6]. In our patient, a confirmatory CT was done, which, along with joint aspiration, confirmed the diagnosis of SCJ septic arthritis. US alone did not prove to be sufficient for diagnosis. CT is also helpful in selecting a surgical strategy by identifying affected surrounding structures [7].

Management of SCJ septic arthritis involves surgical debridement and intravenous antibiotics [5]. Medical management alone in 42% of cases reported by Ross et al. resulted in failure 15% of the time [2]. In a study by Von Glinski et al., all 13 patients were treated with SCJ resection due to the involvement of surrounding structures, abscess formation, and to prevent recurrence of infection due to remaining infected bone and tissue [7]. Song et al. reported that complete SCJ resection resulted in no recurrences, while debridement and antibiotic therapy alone led to recurrence in five of six patients [8]. Our patient underwent SCJ resection and debridement without recurrence of infection.

Empiric intravenous antibiotics, such as oxacillin or cefazolin, should target the most common pathogen isolated, which is *Staphylococcus aureus* (49%). If there are risk factors for methicillin-resistant *Staphylococcus aureus *(MRSA), such as IV drug use, vancomycin should be started instead. The other pathogens isolated in the study by Ross et al. were *Pseudomonas aeruginosa* (10%), *Brucella melitensis* (7%), and *Escherichia coli *(5%). Usually, for uncompleted SCJ septic arthritis, IV antibiotics need to be continued for four weeks or even longer, such as six weeks in cases complicated by osteomyelitis [2].

## Conclusions

We present a case of a young diabetic male patient who initially was diagnosed with a shoulder sprain and concern for a possible gout flare, but was found to have MSSA SCJ septic arthritis on CT scan and joint aspiration. This case highlights the importance of considering SCJ septic arthritis in the differential diagnosis of shoulder pain, given its vague referred symptoms, which can also present as chest pain and clavicular pain. It is important not to overlook the physical exam of the SCJ, as well as recognizing its insidious, often delayed clinical presentation due to its location and indistensibility as a joint space. This can lead to an unfortunate delay in care and treatment, as in our patient, although prompt treatment upon secondary presentation with IV antibiotics and surgical resection resulted in an excellent outcome and successful resolution of infection. Untreated infections can lead to severe complications, including mediastinitis, septic shock, and mortality.
